# Functional Brain Correlates of Upper Limb Spasticity and Its Mitigation following Rehabilitation in Chronic Stroke Survivors

**DOI:** 10.1155/2014/306325

**Published:** 2014-07-03

**Authors:** Svetlana Pundik, Adam D. Falchook, Jessica McCabe, Krisanne Litinas, Janis J. Daly

**Affiliations:** ^1^Neurology and Research Service, Cleveland VA Medical Center, 10701 East Boulevard, Cleveland, OH 44106, USA; ^2^Department of Neurology, Case Western Reserve University School of Medicine, 11100 Euclid Avenue, Cleveland, OH 44106, USA; ^3^Department of Neurology and McKnight Brain Institute, Brain Rehabilitation Research Center of Excellence, Malcom Randall VA Medical Center, University of Florida, 1601 SW Archer Road, Gainesville, FL 32608, USA

## Abstract

*Background.* Arm spasticity is a challenge in the care of chronic stroke survivors with motor deficits. In order to advance spasticity treatments, a better understanding of the mechanism of spasticity-related neuroplasticity is needed.* Objective.* To investigate brain function correlates of spasticity in chronic stroke and to identify specific regional functional brain changes related to rehabilitation-induced mitigation of spasticity.* Methods.* 23 stroke survivors (>6 months) were treated with an arm motor learning and spasticity therapy (5 d/wk for 12 weeks). Outcome measures included Modified Ashworth scale, sensory tests, and functional magnetic resonance imaging (fMRI) for wrist and hand movement.* Results.* First, at baseline, greater spasticity correlated with poorer motor function (*P* = 0.001) and greater sensory deficits (*P* = 0.003). Second, rehabilitation produced improvement in upper limb spasticity and motor function (*P* < 0.0001). Third, at baseline, greater spasticity correlated with higher fMRI activation in the ipsilesional thalamus (rho = 0.49, *P* = 0.03). Fourth, following rehabilitation, greater mitigation of spasticity correlated with enhanced fMRI activation in the contralesional primary motor (*r* = −0.755, *P* = 0.003), premotor (*r* = −0.565, *P* = 0.04), primary sensory (*r* = −0.614, *P* = 0.03), and associative sensory (*r* = −0.597, *P* = 0.03) regions while controlling for changes in motor function. * Conclusions.* Contralesional motor regions may contribute to restoring control of muscle tone in chronic stroke.

## 1. Introduction

Motor rehabilitation is a challenging task especially for individuals who exhibit spasticity along with motor impairment. Spasticity can limit effective practice of coordinated movement and hinder functional recovery and rehabilitation [1–3]. In fact, a more complete restoration of motor function is achieved when spasticity is absent [4]. The obstacle that spasticity creates for upper limb rehabilitation is due to restriction of movement, in opposition to the spastic muscle activity, as in practice of wrist and finger extension when wrist and finger flexors exhibit spasticity. Spasticity burdens a significant portion of patients with chronic motor deficits, secondary to stroke and other types of brain injury. Up to 42% of stroke survivors exhibit abnormal hypertonia [4–8]. This abnormally elevated muscle tone is likely to impact quality of life because it affects many aspects of everyday function, produces pain and discomfort, and prevents normal movements [3, 9].

Spasticity can be improved to some degree. Currently available treatment modalities for spasticity include pharmacological agents (oral preparations, neuromuscular blockade agents) and physical motor therapies (for review, see [10]). Pharmacological agents do not cure spasticity, require continuous redosing, and cause untoward side effects. Some rehabilitation therapies to alleviate spasticity can produce changes in motor function and improve spasticity. These therapies include stretching, strengthening, and electrical and vibratory stimulation [11, 12]. Though these interventions are promising, they produce only partial recovery of normal muscle tone, for some individuals.

Neuroplasticity is involved both in spasticity development following acute central nervous system (CNS) injury and in mitigation of spasticity as result of rehabilitation therapies [13]. Following stroke, diminished cortical modulation of brainstem muscle tone centers leads to disruption of the inhibitory-excitatory balance of the brainstem nuclei [14, 15]. There is also structural and functional reorganization of the supratentorial networks; however, the specific motor behavior-related characteristics of the reorganization are poorly understood. Functional imaging studies described brain activation patterns during passive movement of spastic limb [16–19], but there have been no studies that characterized spasticity-related features during active functional motor tasks. Furthermore, a few studies described changes in brain activation following neuromuscular blockade treatment of hypertonicity [16, 20–23], but it is unknown which changes in brain activation occur in relationship to mitigation of spasticity as result of rehabilitation. Overall, there has been no characterization of the functional brain changes associated with volitional movement-related spasticity caused by stroke or recovery of spasticity in response to rehabilitation. The goal of this study was to test the hypothesis that upper limb poststroke spasticity and the recovery from upper limb poststroke spasticity are associated with distinct changes of activity in the brain regions that control muscle tone, as defined by functional magnetic resonance imaging (fMRI). A secondary purpose was to describe the relationship between spasticity and sensory-motor function for individuals with persistent upper limb deficits after stroke.

## 2. Methods

### 2.1. Participants and Upper Extremity Rehabilitation Protocol

Stroke survivors were enrolled according to the following inclusion criteria: age > 21; history of a single stroke more than 6 months ago; residual upper extremity weakness; at least a trace muscle contraction observed in finger flexors, wrist flexors and extensors, shoulder flexors or abductors, shoulder horizontal abductors, and scapular retractors; and being medically stable and with no contraindications for MRI. Individuals with high upper extremity spasticity (Modified Ashworth Scale ≥ 4) were excluded from the study. Age-matched healthy control subjects were enrolled for fMRI testing. Participants were treated for 12 weeks (5 hours/day, 5 days/week) with upper extremity motor learning therapy. This therapy included learning and practicing of joint movements as close to normal as possible with and without the assistance of robotics and functional neuromuscular electrical stimulation (FES) and by practicing components of functional motor tasks. The motor leaning protocol included practice of functional tasks and of task components, based on functional goals. Closer to normal movement practice was achieved through task practice supported by the use of robotics, FES, and verbal and physical cueing and by task modification. Treatment of spasticity was part of the motor learning protocol and included stretching, joint mobilization, soft tissue massage, upper limb weight bearing exercises, and strengthening of muscles antagonistic to the muscles with spasticity, with the latter being achieved by practicing combined volitional muscle activation practice and FES muscle activation.

### 2.2. Clinical Function Outcome Measures

Modified Ashworth Scale (MAS) grades the degree of hypertonia in individual muscles or muscle groups [24]. The Ashworth Scale score was summated for the following muscle groups: shoulder internal rotators, elbow extensors, elbow flexors, forearm pronators, forearm supinators, wrist flexors, wrist extensors, digit flexors, and digit extensors. The Fugl-Meyer upper limb (FM) coordination scale (range of scores from worst to best, 0 to 66) was used as a measure to characterize impairment level at baseline [25, 26]. The FM wrist/hand (FM W/H) is a measure of coordination of wrist and hand movements, with convergent validity of 0.69 (*P* = 0.001) with FIM Self Care scale and good interrater reliability (ICC = 0.96; [27]). Proprioception was a sum of assessments for elbow, wrist, and 1st, 2nd, and 5th metacarpophalangeal joints and was graded on a 0 to 1 scale (0 = impaired joint position sense, 1 = intact). Light touch sensory function was evaluated using monofilament testing (Semmes-Weinstein monofilament test) [28].

### 2.3. MRI Data Acquisition

MRI was acquired on a Siemens Symphony 1.5 T system using a circularly polarized head coil and an interleaved multislice gradient echoplanar imaging (EPI) sequence. BOLD data acquisition parameters were as follows: in-plane resolution was 3 × 3 mm, repetition time (TR) was 3.87 s, echo time (TE) was 50 ms, flip angle was 90 degrees, and the number of axial slices through the entire cerebrum was 36. For T1 images, in-plane resolution was 1 × 1 mm, TR = 2.16 s, TE = 3.45 ms, and flip angle was 15 degrees. Images were collected in the axial plane.

BOLD protocol was a block design with alternating move and rest periods (10 scans/period; 40 seconds/block); rest/move cycles were repeated 5 times; the paradigm started and ended with a rest block. The movements were performed in a slow, continuous manner, at a rate of 0.2 Hz. The potential for confounding mirror movements in the nontested limb was monitored through the use of an MRI-compatible EMG data acquisition system, and any scans were removed if confounded with unintended muscle activation, according to methods previously described [29]. EMG data were acquired using bipolar surface electromyography (EMG) electrodes that were placed over seven muscles of the opposite arm: anterior deltoid, triceps, biceps, wrist flexors, wrist extensors, finger flexors, and finger extensors (Brain Vision LLC, Morrisville, NC, USA).

We tested brain function during the combined multijoint movement task of grasp preparation, consisting of wrist extension, finger extension, and forearm supination to neutral. The subject's hand was positioned on his/her upper abdomen. Resting position was with forearm pronated, fingers in a relaxed flexed position on the abdomen. A verbal movement command cued the subject either to begin the movement or to rest.

Practice sessions were performed the day before (outside of the MRI department) and on the day of testing (in the MRI room) in order to ensure that the motor tasks were understood and accurately performed. The practice sessions better insured that the subjects performed the task at the level of effort that was as free as possible of mirror movement or muscle activation in the unaffected arm. During fMRI data acquisition, the amount of head motion was <0.4 mm of translation in the *x*, *y*, and *z* directions and <0.4° of rotation. Untoward head and body movements were avoided by the following steps: (1) the head was stabilized with dense sponge materials wedged between the subject's head and the head coil, (2) the torso was stabilized using a strap system, and (3) prepractice of movement occurred on two different occasions.

### 2.4. MRI Data Analysis

MRI data were processed and analyzed using the Statistical Parametric Mapping (SPM5) package (Wellcome Department of Imaging Neuroscience at University College London, UK), along with custom in-house software analysis packages designed by our lab using the MATLAB (The MathWorks, Inc., Natick, MA) technical computing environment.

The fMRI data preprocessing using SPM included the following steps: discarding the initial 2 scans for each rest and move block, slice-timing correction, and head motion corrections. Anatomical and BOLD images were coregistered. Images were normalized to the MNI template. Spatial smoothing was performed with a 6 mm^3^, full width at half maximum Gaussian kernel. Images were right/left flipped in order to align the lesion hemispheres which were all contralateral with the tested arm. Scans that were associated with mirror movements were excluded from the analysis [29].

Individual activation maps were determined by contrasting rest versus move data using *t*-test analysis. A *P* value threshold for calculation of the individual activation map was derived from a *t*-test between rest and move average signal intensity maps of the whole cohort (*P* < 0.05, with correction for multiple comparisons [30]). The product of this comparison was a threshold *P*  value = 0.00062, which was then applied in order to determine brain activation in individual subjects.

Regional fMRI activations were extracted for the following bilateral motor-sensory regions of interest (ROI): (1) primary motor region (M1, Brodmann area (BA) 4); (2) primary sensory region (S1, BA 3); (3) lateral premotor area (LPM); (4) supplementary motor area (SMA) proper (medial portion BA 6 that is posterior to the anterior commissure line) [31]; (5) thalamus; (6) cerebellum. Each ROI was utilized in comparing brain activation before and after upper extremity rehabilitation according to brain activation patterns during the functional motor task. In addition, brain activation measures within each ROI were utilized in investigation of brain activation pattern correlates with the measure of spasticity.

### 2.5. fMRI Measures of Voxel Count and Percent Signal Intensity Change

For each ROI, we calculated the average signal intensity of the active cluster. Percent signal intensity (SI) during the motor task, for each subject, per voxel was calculated as follows:

(1) $$\begin{array}{c} \%\text{SI}v=100*\frac{\text{avgInt}M-\text{avgInt}R}{\text{avgInt}R}, \end{array}$$

where %SI*v* is task-related change in signal intensity in a given voxel, avgInt*M* is average signal intensity of the given voxel across all move scans, and avgInt*R* is average intensity across all rest scans.

### 2.6. Statistical Analysis

Data analysis was performed using SPSS 11.5 software. Within each ROI, Spearman correlation analysis was performed between the spasticity measure and the fMRI signal intensity measure. Missing values were excluded listwise. Data distribution normality was evaluated with Kolmogorov-Smirnov test. For nonnormally distributed data, Wilcoxon signed ranks test was used for pre-/postcomparisons. For investigation of the relationship between treatment response improvement of spasticity and change in brain activation, statistical models were analyzed in order to partial out (i.e., control for) the effect of motor function improvement, so that the relationship between mitigated spasticity and change in fMRI activation was separated from the factor of improved motor function. For this analysis, we used Partial Correlation Analysis; this analysis was conducted in the standard method. Descriptive data were generated for pre-/postvalues, and outliers were identified as beyond the 95% confidence interval; this procedure yielded three outlier data points (subjects) which were then removed for the analysis (i.e., *n* = 20 subjects included in this analysis). The Partial Correlation Analysis was conducted in a series of two steps, as follows: (1) analysis of two separate regression models: specifically (a) motor function with spasticity and (b) motor function with fMRI; (2) then the correlation of the residuals from those two prior models was analyzed in order to obtain a correlation of spasticity improvement with change in fMRI brain activation, separate from the potential confounding variable of gain in FM motor function [32, 33].

## 3. Results

**Figure 1:**
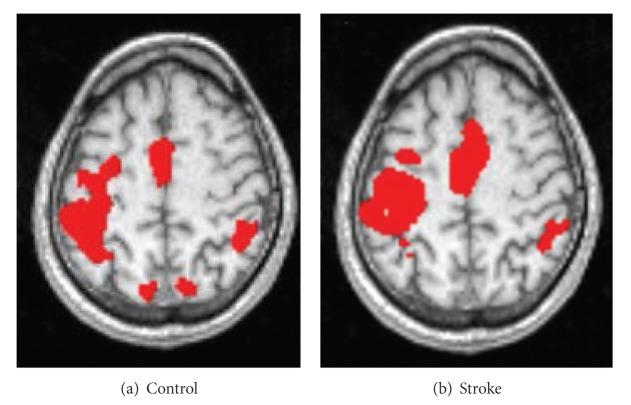
fMRI activation during grasp preparation task: average maps for healthy control group (a) and stroke group (b). Tested arm was contralateral to the hemisphere on the left side of each image.

Subjects' characteristics are shown in Table 1. Healthy control subjects (*n* = 11) were 54.5 ± 12.9 years old; 54% were female. Average fMRI activation maps for both control and stroke groups are shown in Figure 1.

### 3.1. Baseline Characteristics and Correlation between Spasticity and Sensory-Motor Deficits

At baseline, abnormal muscle tone of the upper extremity (MAS > 0) was present for all study subjects. Specifically, spasticity was present in wrist/hand movements in 100% of subjects.

**Figure 2:**
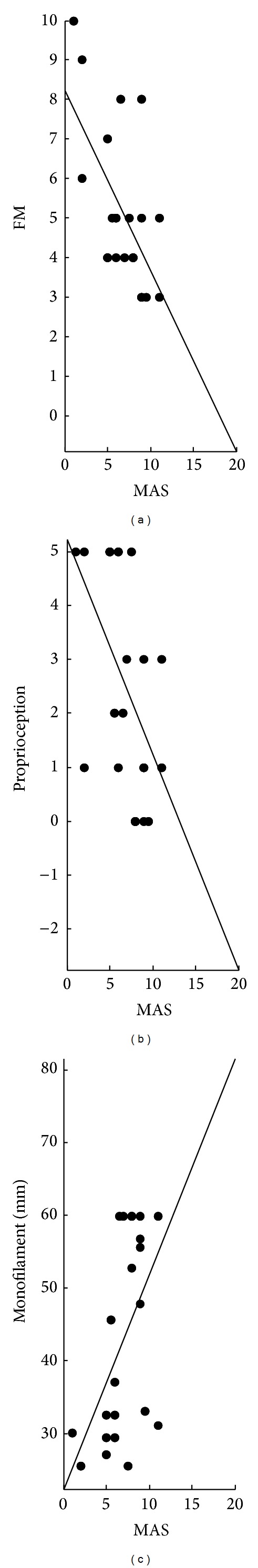
At baseline, greater spasticity according to summated upper limb MAS scores correlated with poorer skilled motor function and greater sensory deficits (MAS—Modified Ashworth Scale; FM—Fugl-Meyer for upper limb).

We found that greater spasticity was correlated with poorer function according to FM score (rho = 0.62, *P* = 0.002) and with greater severe sensory deficits (monofilament test: rho = 0.6, *P* = 0.003; proprioception: rho = 0.6, *P* = 0.003) (Figure 2).

### 3.2. Baseline BOLD Signal Intensity Was Correlated with Spasticity

At baseline, greater spasticity was correlated with higher task-related signal intensity in the ipsilesional thalamus (rho = 0.49, *P* = 0.03).

### 3.3. Correlation of Brain Activation and Spasticity in Response to Rehabilitation: Significant Correlation between Improvement in Spasticity and Change in Task-Related Brain Activation

Following rehabilitation, there was a statistically significant improvement in upper extremity spasticity (*P* < 0.0001; Table 2). The study participants had statistically significant gains in motor function measured with FM total score (*P* < 0.0001; Table 2) and with FM W/H (*P* < 0.0001; Table 2).

**Figure 3:**
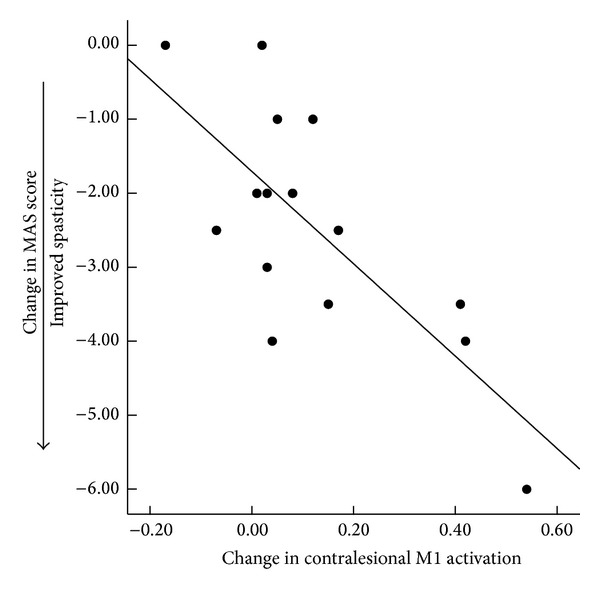
Increase in task-related brain activation correlated with improvement in spasticity.

Mitigation of spasticity was correlated with increased task-related activation in the contralesional M1, LPM, S1, and AS regions (Table 3, Figure 3). This statistically significant relationship was exhibited, while controlling for changes in motor function, according to FM W/H (Table 3).

## 4. Discussion

### 4.1. In Response to Treatment, Improvement in Spasticity Is Associated with Greater Task-Related Brain Activation in the Contralesional Primary Motor Region and Contralesional Lateral Premotor Cortex

To our knowledge, this is the first study to demonstrate a relationship between changes in motor, task-related, brain activity, and upper limb spasticity following a course of physical therapy. Our findings suggest that the contralesional M1 region can have an effect on spasticity of the impaired working limb (i.e., the limb on the same side of the body as the contralesional M1). This is possible because a portion of the nonpyramidal motor tracts that control muscle tone travels in an uncrossed manner from M1 and LPM to the spinal cord. In fact, nonpyramidal corticoreticular tracts are distributed bilaterally [34, 35]. This is in contrast to the pyramidal corticospinal tracts that control volitional movement and are predominantly crossed (approximately 90% of the corticospinal tract fibers that originate from one hemisphere innervate the contralateral upper and lower extremities) [36]. Stroke may have led to permanent damage that prevented reorganization of the ipsilesional nonpyramidal network but spared the contralesional nonpyramidal network. Given this functional anatomy, it is reasonable to consider that the spared contralesional hemisphere could contribute to the recovery of spasticity in an affected limb and that enhanced activation in the contralesional M1 after treatment is consistent with mitigated spasticity. Of note, in contrast to our findings for the contralesional hemisphere, the ipsilesional M1 and LPM regions did not show a relationship with improved spasticity. These findings, together, may reflect improved function due to reorganization of the uncrossed, contralesional, nonpyramidal corticoreticular spinal pathways.

### 4.2. Association of Spasticity with Diminished Somatosensory Function and with Greater Signal Intensity in the Ipsilesional Thalamus during Active Wrist/Hand Movements in Stroke

There is a paucity of information regarding brain function correlates of spasticity after stroke. One study investigated the passive movement task and its associated correlates of spasticity and brain activity. Lindberg et al. [17] characterized brain activity associated with passive upper extremity movements in adults with chronic poststroke spasticity. They reported a correlation between resistance to* passive movement* and brain activity in the primary sensory and motor regions, S1 and M1, that were ipsilateral to the moved upper extremity (i.e., the contralesional hemisphere). Our study extends the literature by providing, for an* active upper limb movement* task, the identification of elevated signal intensity in the ipsilesional thalamus produced by muscle groups that are opposed during movement, by spasticity in the antagonist muscles.

### 4.3. Potential Mechanisms of the Onset of and Recovery from Spasticity

Landmark early basic science studies [37] have described the nature of and potential mechanisms for spasticity onset after stroke and possible spasticity mitigation mechanisms; our results are consistent with the mechanism for abnormal “antigravity postures” (spasticity) in monkeys elucidated in these studies [37]. Specifically, Denny-Brown demonstrated that damage to precentral regions anterior to the primary motor cortex (includes LPM area), as well as primary and secondary parietal sensory regions, is necessary in order to induce spasticity. Lesions limited to the pyramidal corticospinal tract impair deftness and the ability to produce fine finger movements, but lesions isolated to the nonpyramidal corticospinal pyramidal tract do not lead to chronic changes of muscle tone [38]. Of note, the primary motor area (M1) hosts neurons that form both pyramidal and nonpyramidal cortical tracts [34]. The corollary to these classic animal studies is that nonpyramidal neurons of the M1 and LPM regions play a critical role in maintaining normal muscle tone, and their functionality is likely driving spasticity mitigation as a result of rehabilitation in humans.

In addition to the descending, uncrossed, ipsilateral muscle tone control network, deregulation of transcallosal inhibition may also play a role in development of abnormal muscle tone. In a cross-sectional study of brain activation during passive velocity-dependent movement, Lindberg et al. [17] considered that the elevated brain activity in the contralesional hemisphere during passive movements may reflect poststroke alterations of interhemispheric inhibition. In fact, for subjects with moderate to severe motor deficits, a recent study showed that inhibitory repetitive transcranial magnetic stimulation of the contralesional M1 region, combined with physical therapy of the upper limb, led to an improvement of wrist flexor spasticity, while therapy alone did not [39]. Transcallosal neurons originating in the contralesional M1 region may influence activity of the ipsilesional nonpyramidal descending tracts. However, the direct evidence of this potential mechanism is yet to be elucidated. Overall, there is likely to be more than one pathway involved in spasticity after stroke. Further studies will be needed to determine the specific role of the contralesional activity in restoring muscle tone control.

### 4.4. Contrasting Patterns of Pretreatment Brain Activation with Spasticity Present versus Posttreatment Brain Activation with Mitigated Spasticity

After treatment, there was a change in the brain activation patterns associated with spasticity during active movement. That is, at baseline, greater spasticity was associated with elevated activation in ipsilesional thalamus. Although the specific pathways that involve thalamus in regulation of muscle tone may be poorly understood, spasticity-related thalamic activation may be compensatory and maladaptive. A single case report demonstrated that spasticity improved following modulation of thalamic activity with deep brain stimulation for an individual with chronic stroke [40].

In contrast to the baseline condition, at posttreatment, a different brain activation pattern emerged; that is, mitigation of spasticity was associated with enhanced activation in contralesional primary and premotor areas. One explanation of these differences could be that therapy-generated reduction in spasticity may be driven by changes in brain networks unique from maladaptive mechanisms producing the onset of spasticity after stroke.

### 4.5. Potential Effect of the Sensory Network Centers on Mitigation of Spasticity

We found that spasticity correlated with sensory-related clinical and imaging measures both at baseline and following rehabilitation. At baseline, greater spasticity was associated with more severe sensory impairment and higher activation in ipsilesional thalamus. Following rehabilitation, greater improvement in spasticity correlated with higher activation in both primary and secondary sensory regions. A few rehabilitation studies imply, but do not define, a connection between sensory input and spasticity. The indirect evidence of such connection is derived from studies that report two types of findings. First, some found that greater spasticity was correlated with diminished activation in sensory regions [41]. Second, some found that spasticity treatment was associated with changes in sensory network function [16, 19, 42]. Peripheral sensory treatments improved spasticity and changed function in sensory networks [19, 42]. Furthermore, treatment of spasticity increased activation in the contralesional secondary sensory cortex and in bilateral primary sensorimotor areas during a passive movement treatment [16]. These studies and our results, taken together, support the important role of the sensory system in spasticity mitigation.

## 5. Conclusion

This study identified brain regions that may be related to improvement in spasticity as a result of rehabilitation therapies. Further characterization of the role of these cortical regions in mitigation of spasticity could guide us in development of novel interventions that will improve rehabilitation outcomes. Interventions currently available to reduce spasticity are limited, temporary, and have significant side effects. Our findings suggest a direction of inquiry that may lead to development of new antispasticity interventions to improve quality of life and functional recovery after stroke.

## Figures and Tables

**Table 1:** Subject characteristics.

	Stroke subjects *n* = 23
Age in years, mean (std. dev.)	56.3 (12.8)
Female (%)	41
Stroke hemisphere (% left)	55%
Stroke type (% ischemic)	88.6%
Years since stroke	1.8 (1.1)
Lesion location *n* (%)	
BG/IC	7 (30%)
Pons	2 (8.6%)
Frontal lobe	1 (2.3%)
Frontal/parietal lobes	3 (13%)
Frontal lobe/BG/IC	3 (13%)
Frontal/parietal lobes/BG/IC	5 (21.7%)
Frontal/parietal/temporal lobes/BG/IC	2 (8.6%)
Medical history	
DM	17.4%
HTN	52.2%
Heart disease	21.7%
Smoking	56.5%

**Table 2:** Gains in muscle function outcome measures from pre- to postrehabilitation.

Outcome measure	Prerehab.	Postrehab.	*P* value*
Fugl-Meyer upper limb (points, mean (SD))	22.2 (8.7)	33.3 (10.4)	<0.0001
Fugl-Meyer wrist/hand (points, mean (SD))	6.34 (3.2)	9.09 (3.4)	<0.0001
Modified Ashworth (points, median (IQR))	7 (4)	4.5 (4.5)	<0.0001

*Wilcoxon signed ranks test.

**Table 3:** In response to treatment: correlations between reduction in spasticity and changes in task-related brain activation with and without adjusting for changes in motor function according to FM wrist/hand scores. Shown here are correlations >0.45.

Hemisphere	ROI	Bivariate correlations *r* (*P* value)	Partial correlations controlling for FM W/H *r* (*P* value)
Contralesional	M1	−0.745 (0.002)	−0.755 (0.003)
	LPM	−0.522 (0.05)	−0.565 (0.04)
	S1	−0.571 (0.03)	−0.614 (0.026)
	AS	−0.529 (0.05)	−0.597 (0.03)

M1: primary motor area, LPM: lateral premotor region, S1: primary sensory, and AS: associative sensory.
